# Retrospective matched case–control comparison of Totally Robotic Sleeve Gastrectomy (RSG) during the learning curve with Laparoscopic Sleeve Gastrectomy (LSG): why is operative time different?

**DOI:** 10.1007/s13304-025-02087-3

**Published:** 2025-01-15

**Authors:** Antonio Vitiello, Giovanna Berardi, Pietro Calabrese, Maria Spagnuolo, Fabrizia Calenda, Giuseppe Salzillo, Roberto Peltrini, Vincenzo Pilone

**Affiliations:** 1 Advanced Biomedical Sciences Department, Naples “Federico II” University, AOU “Federico II” - Via S. Pansini 5, 80131 Naples, Italy; 2 Clinical Medicine and Surgery Department, Naples “Federico II” University, AOU “Federico II” - Via S. Pansini 5, 80131 Naples, Italy; 3 Public Health Department, Naples “Federico II” University, AOU “Federico II” - Via S. Pansini 5, 80131 Naples, Italy

**Keywords:** Robotic surgery, Pain, Sleeve gastrectomy, Learning curve, Robotic bariatric surgery

## Abstract

Robotic approach is slowly rising in metabolic surgery, and laparoscopy is still considered the gold standard for Sleeve Gastrectomy. Aim of our study was to assess and compare outcomes of RSG through a matched comparison with LSG. Retrospective search of prospectively maintained database of our surgical department was carried out find all consecutive patients who underwent RSG from April 2023 to August 2024. Each subject who underwent RSG was matched one-to-one with a patient treated with LSG in the same period. Operative time (docking + console time for the robotic procedures), length of stay, need for rescue drugs, and perioperative complications were recorded calculated and compared. A total number of 50 patients (25 RSG and 25 LSG) were included in the present analysis. Operative time in the LSG group was significantly shorter than in the RSG group (57.8 ± 12.3 VS 80.6 ± 16.6 min, *p* < 0.01), but it was comparable to console time (57.8 ± 12.3 VS 56.9 ± 19.6, *p* = 0.85). Mean docking time was 23.7 ± 11 min. Length of stay, readmissions, conversion to laparoscopy/open surgery, early complications, and rescue drugs administration were comparable between the two groups. Age, sex, and BMI were not good predictors neither of laparoscopic nor robotic operative time. RSG during the learning curve proved as safe as LSG, but it was associated with longer operative time due to the duration of the docking step. Operation length may become comparable once the learning curve plateau is reached. Age, BMI, and sex are not good criteria of choice between the two approaches.

## Introduction

Laparoscopic Sleeve Gastrectomy (LSG) is the most common metabolic procedure worldwide [1], and it represents more than half of all bariatric interventions in many countries [2].

Due to the laparoscopic feasibility of LSG, bariatric surgeons are still strictly bonded to the laparoscopic side of mini-invasive surgery. According to the last IFSO Report [3], 23% of all primary metabolic interventions were performed robotically in the U.S. On the contrary, despite the high number of metabolic procedures that are performed annually worldwide, rate of robotic surgery is dramatically low in many European countries, such as Italy (0.6%), France (0%), or Sweden (0%).

This gap with other surgical specialties (colorectal, abdominal wall, gynecological, urological, and thoracic) needs to be filled [4].

The slow rise of robotic surgery [5] in the MBS field is even more unjustified considering that this approach may help to overcome technical challenges such as small intraabdominal space, increased liver size, abundant intra-abdominal fat, and a thick subcutaneous tissue [6, 7].

Moreover, due to the absence of a standardized technique, some steps, like the stapling part, are sometimes performed laparoscopically.

To obtain significant advancements over laparoscopic surgery, fully Robotic Sleeve Gastrectomy (RSG) with all the surgical steps carried out using robotic instruments should be standardized.

In our institution, after the use of a hybrid technique for the first patients, we rapidly switched during our learning curve to the fully RSG.

Aim of this study was to retrospectively assess and compare outcomes of RSG through a matched comparison with LSG with special regard to operative time, rate of perioperative complications and length of stay.

## Methods

Retrospective search of prospectively maintained database of our surgical department was carried out to find all consecutive patients who underwent RSG from April 2023 to August 2024. Indication for metabolic bariatric surgery (MBS) in our center is age between 18 and 60 years, and BMI > 35 kg/m2 or > 30 with an obesity-related disease [8]. Subjects with a previous history of bariatric or abdominal surgery, and those with signs of severe Gastro-Esophageal Reflux or Hiatal Hernia > 2 cm at the Upper GI endoscopy were excluded. Each subject who underwent RSG was matched one-to-one with a patient treated with LSG in the same period with correspondent sex, preoperative BMI (± 2 unit), and age (± 2 year). Collected data at baseline were sex, age, and body mass index (BMI).

Operative time (docking + console time for the robotic procedures), length of stay, conversion to open or laparoscopy, reinterventions, readmissions, and intraoperative and early (< 30 days) postoperative complications (bleeding, leak, and stenosis) were recorded in a Microsoft Excel 365™ datasheet. Rates of rescue antiemetics and painkillers were also calculated and compared. Written informed consent was obtained from each participant.

Learning curve phase was assessed according to recent literature [9].

### Robotic surgical technique

**Fig. 1:**
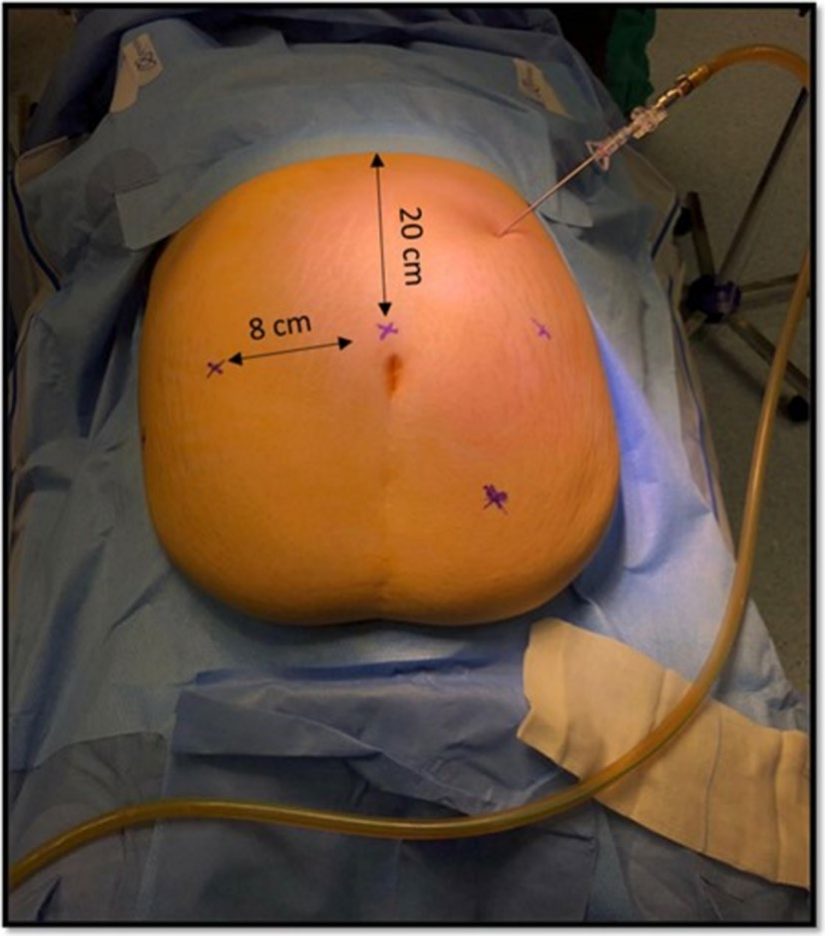
Distance between trocars

The patient was placed in a supine position with open legs; first assistant stood between them. CO^2^ pneumoperitoneum was obtained through Veress needle positioned in the Palmer’s point (Fig. 1). A first 8 mm trocar was inserted on the midline 15–20 cm from the xiphoid process with an open technique.

**Fig. 2:**
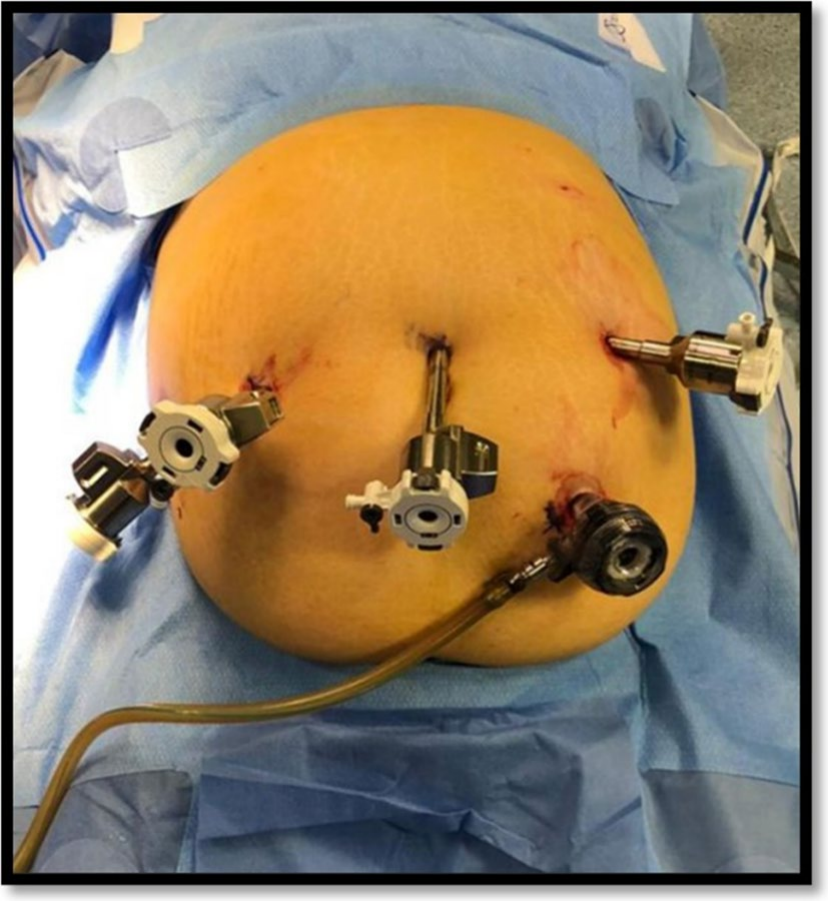
Trocars placement

Other 3 robotic trocars (8 mm) were placed on the same horizontal line with an 8-cm distance between them. An accessory 12-mm laparoscopic port was placed in the left flank (Fig. 2).

Da Vinci XI Robot was docked from the left side of the patient. Robotic arms were equipped with the following devices:

• arm 1 – Grasper (Cadiere Forceps^®^ or Prograsp Forceps^®^).

• arm 2 – Grasper (Bipolar Fenestrated Forceps^®^ or Bipolar Fenestrated Maryland®), which will be replaced in some phases of the operation by the robotic stapler (Sureform Stapler^®^).

• arm 3 –30° robotic camera;

• arm 4 –High Energy Device (Vessel Sealer^®^).

**Fig. 3:**
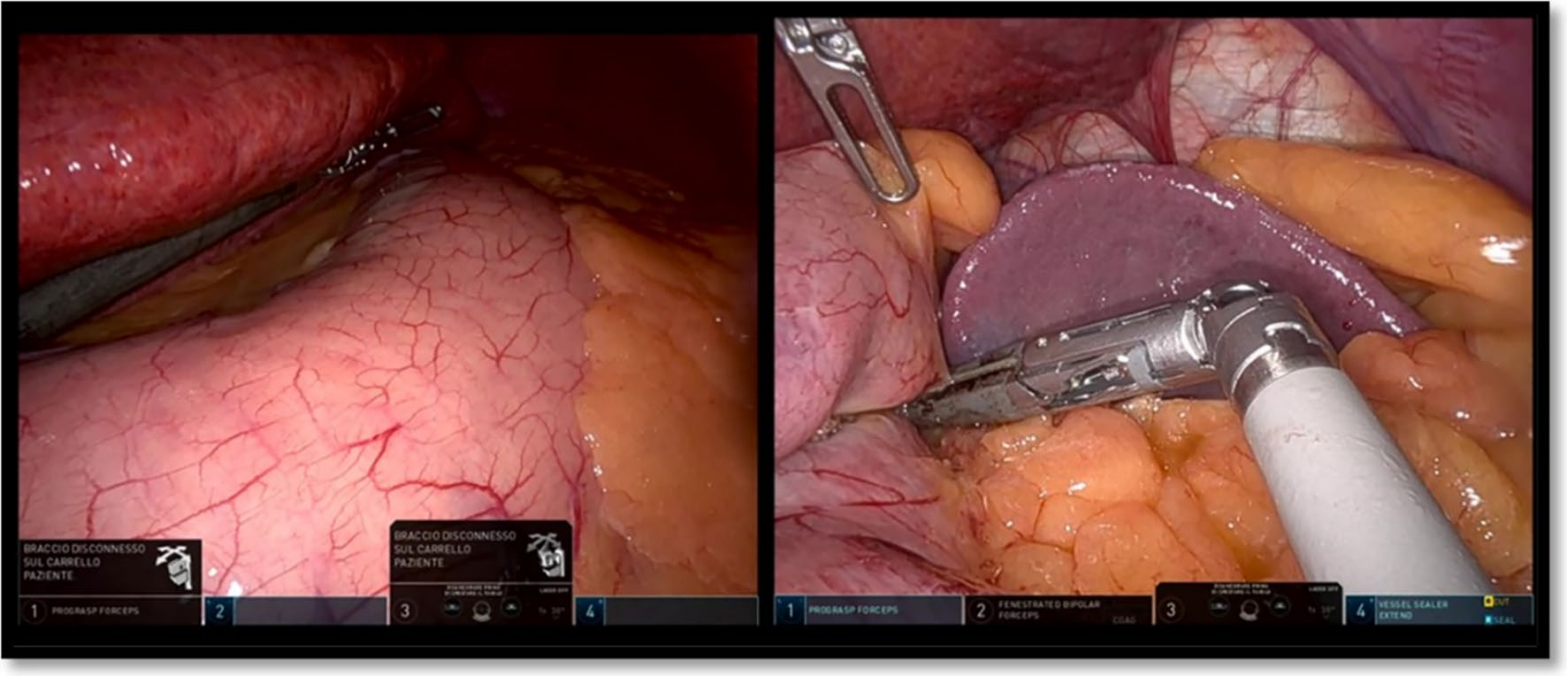
Approach to the short gastric vessels using the grasper in arm 1 to lift the fundus

The grasper in arm 1 was used as liver retractor for almost all the procedure; during the short gastric vessel dissection, it was also used to lift the fundus of the stomach (Fig. 3).

**Fig. 4:**
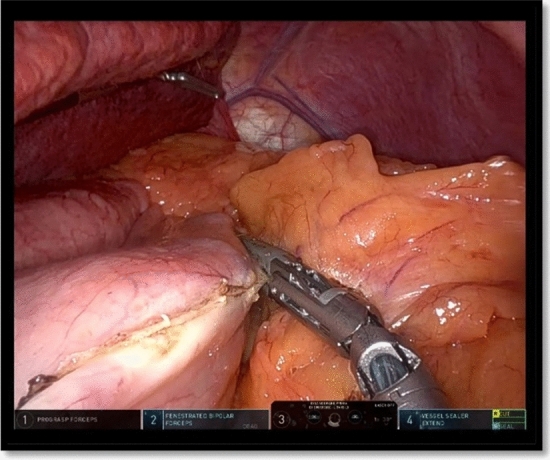
Opening of the gastrocolic ligamente

The operation started with the access to the omental sac and the dissection of the greater omentum along the greater curvature starting 4 cm from the pylorus. This part was performed using the grasper in arm 2 and the vessel sealer in arm 4 (Fig. 4).

After the visualization of the left crus, a 36 French bougie was inserted into the stomach till the prepyloric portion of the antrum. The grasper in arm 2 was replaced with the robotic stapler.

**Fig. 5:**
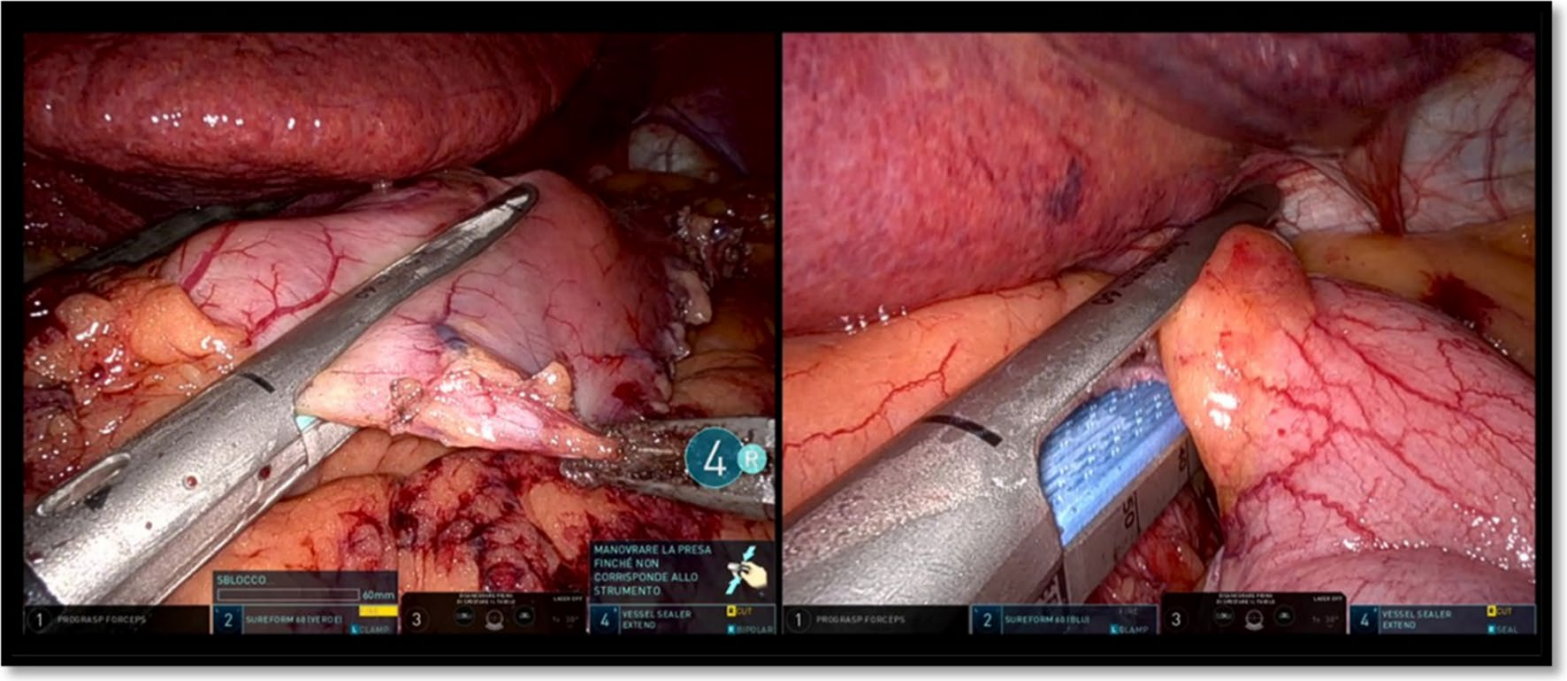
Vertical gastrectomy

Using the vessel sealer to stretch the stomach laterally, a vertical gastrectomy was carried out along the bougie with green cartridges for the antrum and blue ones for the body and the fundus (Fig. 5). Metallic clips were applied in case of bleeding spots on the staple line.

After a negative blue test, the remnant was extracted through the accessory port and a drain was left along the staple line.

### Laparoscopic surgical technique

Five trocars approach (3 × 12 mm, 2 × 5 mm) was used. Gastrectomy started 4 cm from the pylorus over a 36French bougie. An average of 5 cartridges (green for the antrum, yellow for the body, and blue for the fundus) of a 60 mm laparoscopic stapler were used. Last cartridge was fired 1 cm lateral to the esophagogastric junction. Reinforcement was not routinely applied, but in all patients, the staple line was carefully inspected, and metallic clips were applied in case of bleeding spots.

### Perioperative care

No nasogastric tube was left at the end of the procedure, while drain is routinely positioned along the staple line. No abdominal wall block or intraoperative injection of local anesthetics was performed. Postoperative analgesia was provided with a continuous intravenous administration of ketorolac 90 mg, ondansetron 8 mg, and oxycodone 10 mg through an elastomeric infusion pump. All patients were allowed to assume intravenous Paracetamol 1 g or metoclopramide 10 mg as rescue drugs for pain and nausea/vomiting, respectively. A liquid diet was started on postoperative day 1, and asymptomatic patients were discharged from postoperative day 2 onwards.

### Statistical analysis

Data are expressed as mean ± SD for continuous variables and as proportion or percentage in case of categorical ones. Continuous and categorical variables were compared using the t test and Chi-square, respectively. Fisher or Yates correction were used instead of Chi-square when appropriate. Linear regression and R-squared coefficient between operative time (dependent variable) and BMI or age (independent variables) were also calculated. Significant p value was set below 0.05.

## Results

A total number of 50 patients were included in the present analysis. In the study period, 26 RSGs were performed at our center, but 1 patient was excluded from the analysis due to previous abdominal surgery. Twenty-five patients were eventually included in our research, and a control group of 25 individuals that had undergone LSG was obtained through above-mentioned matching criteria.

**Fig. 6:**
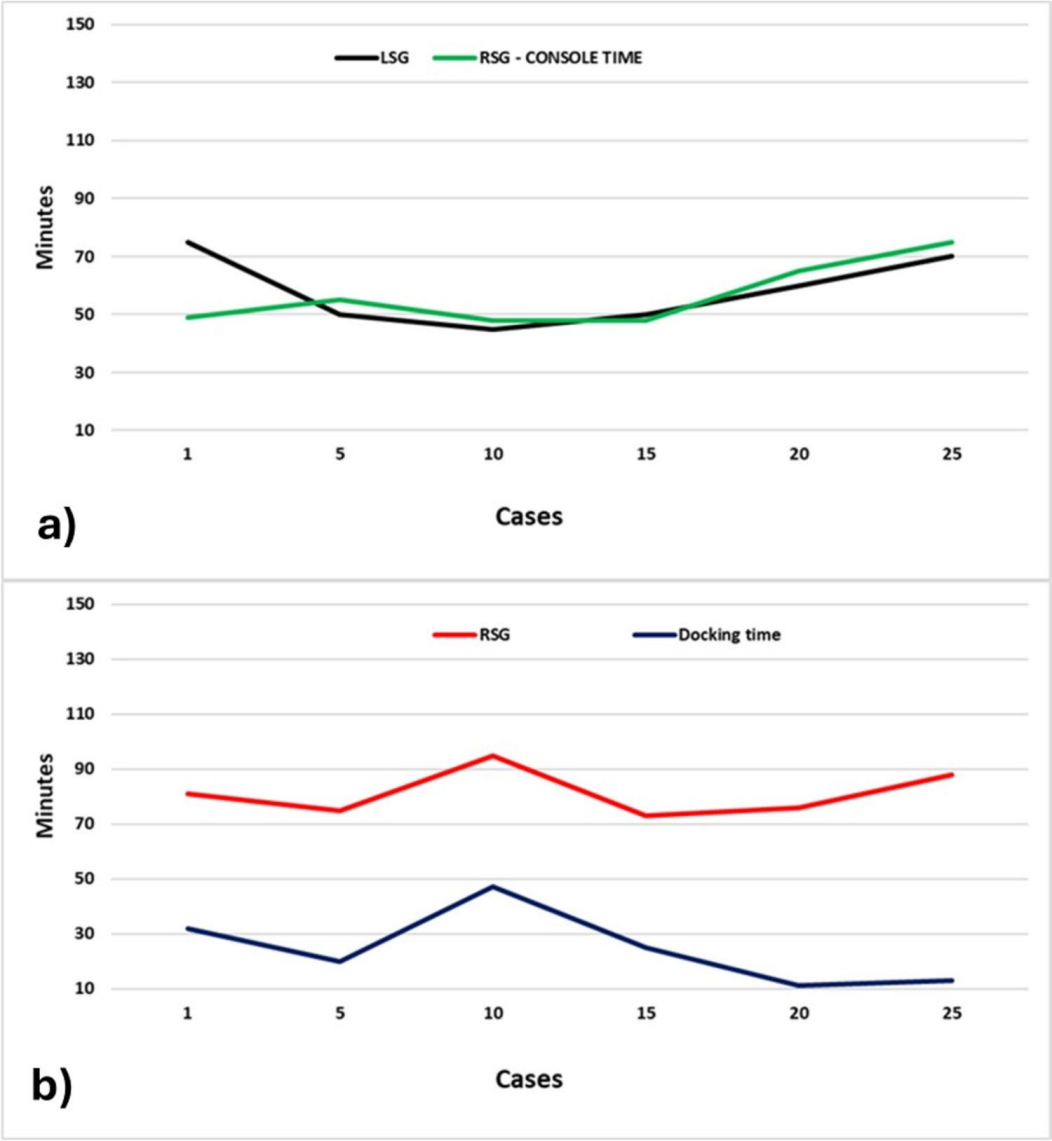
Operative time

Preoperative demographics (age, sex, and BMI) were comparable between the two groups, as shown in Table 1. Operative time in the LSG group was significantly shorter than in the RSG group (57.8 ± 12.3 VS 80.6 ± 16.6 min, *p* < 0.01), but it was comparable to console time (57.8 ± 12.3 VS 56.9 ± 19.6, *p* = 0.85). Mean docking time was 23.7 ± 11 min (Fig. 6).

**Table 1:** Baseline demographics of the two groups

	All patients *(N* = *50)*	RSG *(N* = *25)*	LSG *(N* = *25)*	*P* value
Age (Years)	35 ± 11.5	34.9 ± 11.5	35.2 ± 11.7	*0.93*
BMI (Kg/m^2^)	42.8 ± 5.2	42.7 ± 5.1	42.8 ± 5.3	*0.94*
Sex (F)	32 (64%)	16 (64%)	16 (64%)	*1*
Previous abdominal surgery	0 (0%)	0 (0%)	0 (0%)	*1*

*RSG*  robotic sleeve gastrectomy, *LSG* laparoscopic sleeve gastrectomy, *BMI* body mass index

Length of stay, readmissions, conversion to laparoscopy/open surgery, early complications, and rescue drugs administration were comparable between the two groups, as shown in Table 2. One leak and two bleedings occurred after RSG, but they were all treated with conservative treatment. One patient was readmitted after RSG due to vomiting, but she was successfully discharged after IV infusion of metoclopramide and fluids.

**Table 2:** Peri- and post- operative outcomes in the two groups

	All patients *(N* = *50)*	RSG *(N* = *25)*	LSG *(N* = *25)*	*P* value
Operative time (mins)	69.2 ± 18.5	80.6 ± 16.6	57.8 ± 12.3	< 0.01
Lenght of stay (days)	3.6 ± 1.1	3.9 ± 1.4	3.4 ± 05	0.08
Conversion to laparoscopy	1 (2%)	1 (4%)	0 (0%)	1
Conversion to open	0 (0%)	0 (0%)	0 (0%)	1
Readmissions	1 (2%)	1 (4%)	0 (0%)	1
Leak	1 (2%)	1 (4%)	0 (0%)	1
Bleeding	2 (2%)	2 (8%)	0 (0%)	0.5
Stricture	0 (0%)	0 (0%)	0 (0%)	1
Patients requiring rescue painkillers	11 (22%)	6 (24%)	5 (20%)	0.7
Patients requiring rescue antiemetics	18 (36%)	9 (36%)	9 (36%)	1

*RSG* robotic sleeve gastrectomy, *LSG* laparoscopic sleeve gastrectomy

Operative times were constantly longer in male patients, but without statistical significance (Table 3).

**Table 3:** operative times according to sex

	All patients *(N* = *25)*	Females *(N* = *16)*	Males *(N* = *9)*	*P* value
Robotic Operative time	80.6 ± 16.6	78.1 ± 18.7	84.9 ± 11.7	0.7
Docking Time	23.7 ± 11	22.4 ± 9.2	25.9 ± 13.9	0.1
Console Time	56.9 ± 19.6	55.8 ± 22.6	59 ± 13.3	0.6
Laproscopic Operative Time	57.8 ± 12.3	55.9 ± 13.8	61.1 ± 8.9	0.3

**Fig. 7:**
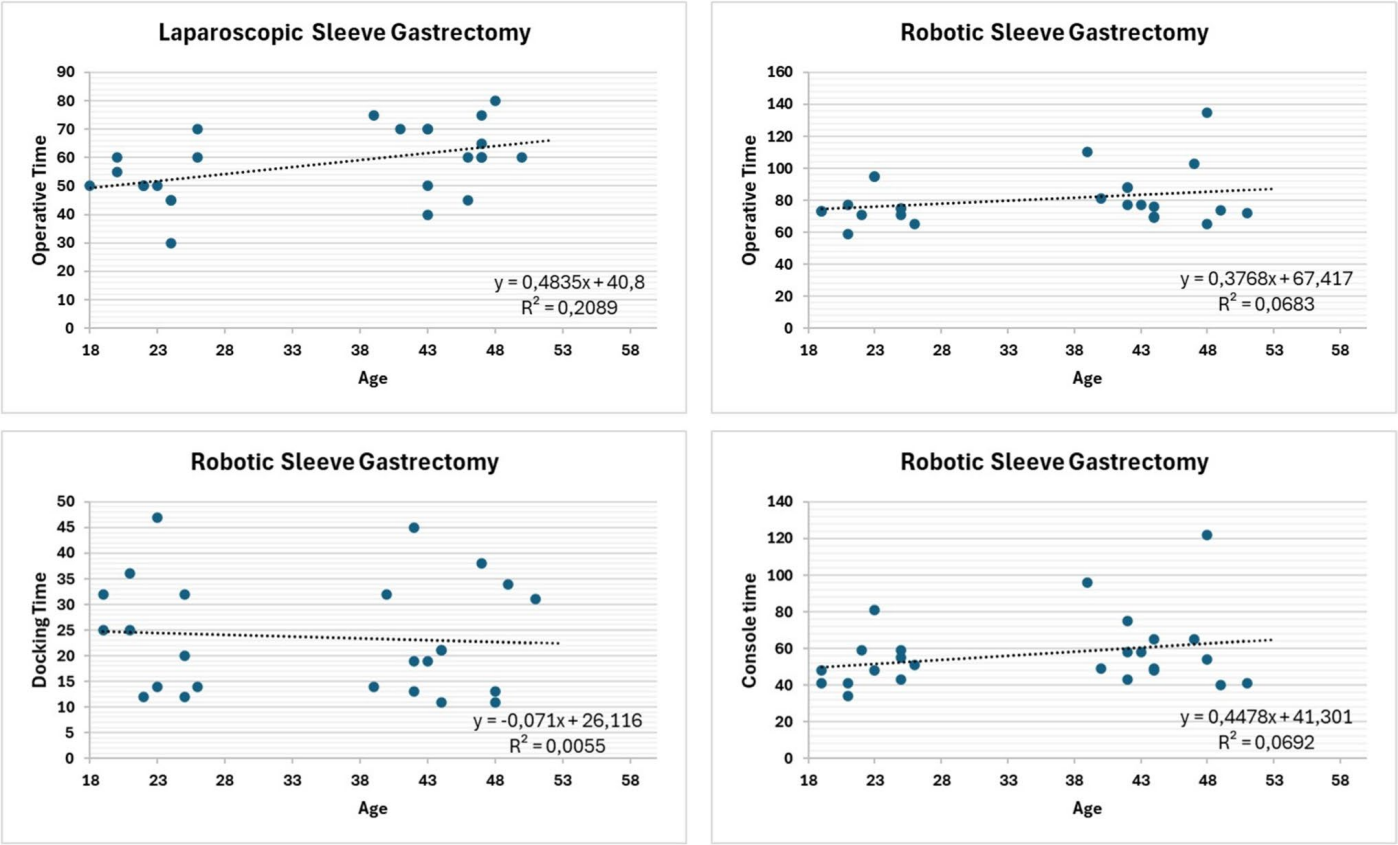
Linear regression between operative time and age

**Fig. 8:**
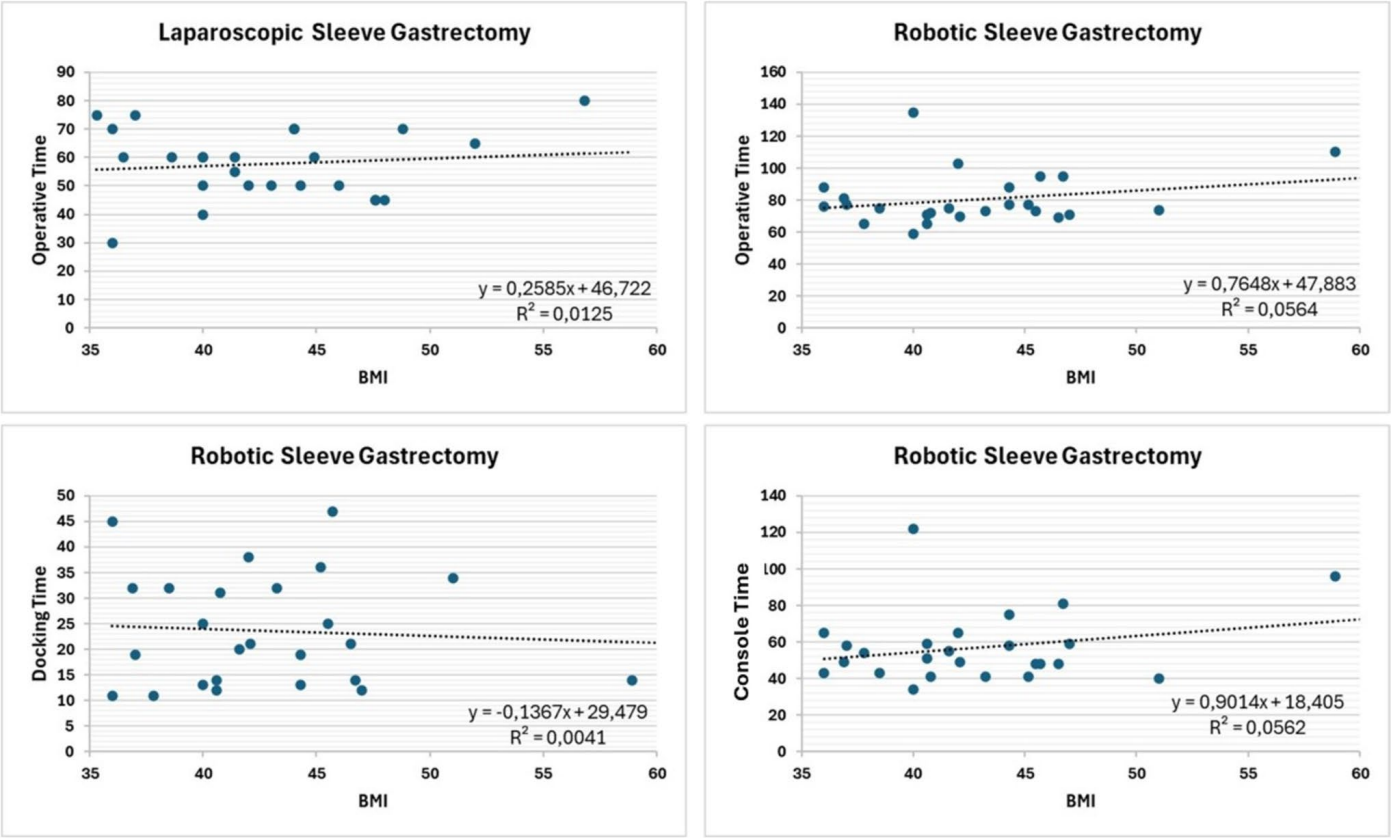
Linear regression between operative time and BMI

Linear regression showed that neither BMI nor age was good predictor of laparoscopic and robotic operative times (Figs. 7 and 8).

## Discussion

Despite robotic surgery is gradually spreading worldwide, many doubts persist regarding its use for metabolic procedures. Main objections against robotic approach are longer operative times, higher costs and no benefits for the patients’ recovery when compared to laparoscopy. Indeed, according to the 2015–2018 Metabolic and MBS Accreditation and Quality Improvement Program (MBSAQIP) database, duration of LSG was shorter than RSG (102.58 ± 46 vs. 73.38 ± 36; *P* < 0.001)[10].

Similarly, an analysis [11] of the 2020 MBSAQIP registry Public Use File (PUF) concluded that robotic-assisted (RA) approach in metabolic and MBS remains controversial because of higher rates of transfusions after RA-Sleeve Gastrectomy. Another study [12] on the 2015–2016 MBSAQIP PUF comparing outcomes in the conventional laparoscopic versus RA revisional MBS (RBS) showed that RBS was associated with longer operative duration and higher rates of complications.

This initial evidence seemed to demonstrate that laparoscopy should continue to be the surgical approach of choice for sleeve gastrectomy [13]. However, recent articles have started reporting encouraging results in favor of RSG. Sebastien et al. have discovered that matched analysis [10] of the 2015–2018 MBSAQIP database shows that postoperative bleeding and blood transfusion are significantly reduced with the robotic platform, and after correcting for all factors including operative time, the RA approach is associated with better postoperative outcomes.

Another matched analysis [14] compared adverse outcomes within 30 days for the 2015–2018 and 2019–2021 MBSAQIP database. Despite this article confirmed higher complications and longer operation time with the RSG, it also demonstrated that leak rate and operation length decreased with surgeon experience and evolution of robotic stapling. Indeed, while the SureForm stapler, an Intuitive Surgical proprietary device, may now be more consistently used, in the past a hybrid technique involving laparoscopic stapling was often performed. In the data of the MBSAQIP database, there is also a lack of information about the use of reinforcements which can affect staple line complications. Moreover, this study has also suggested that there might be a learning curve effect that correlates with worse outcomes in the early reports of RSG.

A recent study using the PINC AI Healthcare Database [15] demonstrated that when stapler type used is accounted for, patient outcomes following RSG and LSG are equivalent. As said before, this finding was probably related to the greater experience with the RSG, which grew in the U.S., from 15% in 2019 to 25% in 2021 with an absolute 27​% increase in robotic stapler utilization for RSG.

Our results are mainly consistent with the published literature. Operative time of the RSG was significantly longer than LSG, but we noted that main difference was due to the docking time. This finding is even more interesting considering that our operative time for RSG met internationally accepted standards, while the docking time was longer than expected [16]. Once the docking step duration is reduced, the discrepancy between two approaches may reduce.

Interestingly, preoperative demographics did not affect the prolongation of the procedure; surgeons have a shared opinion that male individuals and higher BMI are associated with longer times, but this was not proven by our data.

However, despite longer duration of robotic surgery, rescue drugs utilization demonstrated that postoperative nausea and vomit were similar to laparoscopy, and also length of stay was not lengthened.

Even if the only patients of postoperative leak and bleeding of our cohort occurred in the robotic group, rates of complications were comparable. This finding demonstrates the safety of RSG, even during the learning phase, since all RSGs were performed by the first author at the beginning of his robotic experience, while all LSGs were performed by surgeons with a case series > 100 [17].

Our experience with RSG has also led us to consider several advantages of the robotic approach that are often overlooked. First, the articulation of the tip and the 3D view undoubtedly make it easier to perform the procedure; the long jaw of the vessel sealer grants a quicker and safer sealing of the gastric vessels, while the articulation of the stapler helps to avoid twists and turns of the gastric tube^16^. Second, ergonomics is not a negligible benefit [18], since bariatric surgeons often perform several procedures in the same day. Another real advantage of robotic is the potential use of double console, which allows a direct control of the proctor who can easily take over in case of intraoperative complications.

Another point frequently raised against the robotic approach for the SG is that the technique does not involve stitching or anastomosis, which may more obviously benefit from the DaVinci use. This opinion does not consider that more and more frequently SG is performed concomitantly with other interventions, such as abdominal hernia repair and/or cholecystectomy. In all these simultaneous procedures, the use of the robot is certainly beneficial.

This is particularly interesting for the treatment of patients with severe obesity suffering from Gastroesophageal Reflux Disease (GERD) and/or Hiatal Hernia (HH). A recent article [19] has reported that Robotic concurrent MBS and HH repair leads to similar overall clinical outcomes as the laparoscopic approach despite longer operative times.

### Strength and limitations

This is a retrospective study including 25 patients per arm. However, the matched analysis reduces preoperative confounders and learning curve for RSG requires only 5 consecutive interventions [20].

## Conclusion

RSG was associated with longer operative time than LSG due to the duration of the docking step. Despite higher complication rate, RSG proved as safe as LSG during the learning curve. Even if laparoscopy remain the gold standard for sleeve gastrectomy, prospective trials may demonstrate comparable outcomes with RSG, including operation length, when the learning curve plateau is reached.

Age, BMI, and sex did not significantly influence robotic or laparoscopic operative time; thus, they should not be considered as criteria of choice between the two approaches.

## Data Availability

The data that support the findings of this study are available on request from the corresponding author.
